# Loss of control eating with and without the undue influence of weight or shape on self-evaluation: evidence from an adolescent population

**DOI:** 10.1186/s40337-014-0031-1

**Published:** 2014-10-23

**Authors:** Carmel Harrison, Jonathan Mond, Caroline Bentley, Kassandra Gratwick-Sarll, Elizabeth Rieger, Bryan Rodgers

**Affiliations:** Research School of Psychology, Australian National University, Canberra, Australia; Department of Psychology, Macquarie University, Sydney, Australia; Australian Demographic and Social Research Institute, Australian National University, Canberra, Australia

**Keywords:** Binge eating disorder, Loss of control eating, Diagnostic criteria, Overvaluation, Adolescents

## Abstract

**Background:**

The overvaluation of weight and/or shape (“overvaluation”), a diagnostic criterion for anorexia nervosa and bulimia nervosa, is increasingly supported for inclusion in the Diagnostic and Statistical Manual of Mental Disorders 5th Edition (DSM-5) criteria of binge eating disorder (BED). However, current evidence has been largely confined to adult populations. The current study aims to examine the status of overvaluation among adolescents with loss of control (LOC) eating recruited from a large, population-based sample.

**Method:**

Subgroups of female adolescents – LOC eating with overvaluation (n = 30); LOC eating without overvaluation (n = 58); obese no LOC eating (“obese control”) (n = 36); and “normal-weight control” (normal-weight, no LOC eating) (n = 439) – recruited from secondary schools within the Australian Capital Territory (ACT) were compared on measures of eating disorder psychopathology, general psychological distress and quality of life.

**Results:**

Participants in the LOC eating with overvaluation subgroup reported significantly higher levels of eating disorder psychopathology than all other groups, while levels did not differ between participants in the LOC eating without overvaluation and obese control subgroups. On measures of distress and quality of life there were no significant differences between LOC eating with and without overvaluation subgroups. Both reported significantly greater distress and quality of life impairment than normal-weight controls. LOC eating with overvaluation participants had significantly higher levels of distress and quality of life impairment than obese controls, whereas scores on these measures did not differ between LOC eating without overvaluation and obese control subgroups.

**Conclusion:**

The results suggest that the presence of overvaluation among adolescents with LOC eating indicates a more severe disorder in terms of eating disorder psychopathology, however may not indicate distress and disability as clearly as it does among adults with BED.

## Background

The overvaluation of weight and/or shape (“overvaluation”) is described as a dysfunctional system of self-worth, such that the individual evaluates his or her self-worth based primarily or solely on their weight and/or shape [1]. Findings from both community and clinical samples in adults suggest that, among individuals with binge eating disorder (BED) or variants of BED, overvaluation indicates a significantly more severe disorder in terms of eating disorder symptomatology, comorbid psychopathology, and psychosocial impairment [2-5]. Conclusions from such findings suggest that overvaluation should be incorporated among Diagnostic and Statistical Manual of Mental Disorders 5th Edition (DSM-5) criteria for BED, either as a diagnostic specifier or a diagnostic criterion, inline with the other formal eating disorders anorexia nervosa and bulimia nervosa [6-8]. Concerns have been expressed that the absence of overvaluation in the diagnosis of BED may hinder both clinical practice and epidemiological research [9].

A conspicuous limitation of the existing literature however, is that it is almost entirely confined to adult populations [4,5,7]. Given that binge eating is common among children and adolescents, and given the need to develop classification schemes that take in to account eating disturbances across the lifespan, this is problematic [10]. Further, it is increasingly recognised that the DSM-5 definition of binge eating, which requires both consumption of an objectively large amount of food and loss of control over eating, may be problematic in children and adolescents, such that loss of control over eating – but not the amount of food consumed – is key in this population [11,12]. Hence, an alternative diagnostic criterion for BED in children and adolescents, in which the term loss of control (LOC) eating is used in preference to binge eating, has been suggested [11,12].

To our knowledge, only two studies have examined the status of overvaluation in young people with LOC eating [13,14]. Hilbert and Czaja found, in a community based study of children (aged 8–13 years), that overvaluation successfully identified children experiencing high recurrent LOC eating as a more severe presentation, in terms of eating disorder psychopathology, depressive symptoms and behavioural problems than all other groups [13]. Further, overvaluation aided in delineating eating defined as “normal”, and LOC eating among children, thereby enhancing the specificity of classification [13]. Goldschmidt et al. compared overweight youth with LOC eating with ("moderate") and without ("minimal or no") overvaluation, along with comparison groups of overweight youth with no LOC eating with and without overvaluation, on measures of eating disorder psychopathology, behavioural problems and self-esteem [14]. Youth with LOC eating and overvaluation reported greater eating disorder symptomatology than all other groups, whereas youth with LOC eating without overvaluation were comparable to the overweight comparison group of no LOC eating, but reporting overvaluation. Further, youth reporting LOC eating with overvaluation experienced lower self-esteem than the overweight controls without overvaluation, whilst no other significant differences were found between groups on this measure. Both youth experiencing LOC eating with and without overvaluation reported significantly more behavioural problems than overweight controls regardless of overvaluation status. However, overvaluation did not distinguish between LOC eating subgroups on outcome measures of self-esteem and behavioural problems. The authors concluded that overvaluation failed to indicate a more severe symptomatology in adolescence [14].

One reason why anticipated differences may not have emerged in Goldschmidt et al.’s study, is that LOC eating was deemed to be present if participants reported one or more episodes of this behaviour in the past three months, as opposed to the DSM-5 binge frequency criterion for BED of one or more episodes per week [14]. Given the use of this relatively liberal criterion for “regular LOC eating”, it is possible that LOC eating in Goldschmidt et al.’s study was not severe enough for differences between groups to emerge. The use of overweight – as opposed to obese – comparison groups could have further minimised group differences. Finally, Hilbert and Czaja’s study included youth up to age 13 years, thus Goldschmidt et al.’s study is the only study to our knowledge, investigating overvaluation in BED and variants of BED within adolescents aged 13–18 years. Given recent interest in developing diagnostic criteria for eating disorders that are applicable across the lifespan [10], and observed differences between children and adults in terms of overeating behaviour [11,12], the lack of research addressing the status of overvaluation among adolescents with LOC eating is problematic.

The aim of the present study was, therefore, to examine the status of overvaluation in relation to LOC eating in an adolescent sample. Specifically, we sought to compare levels of eating disorder psychopathology, general psychological distress and impairment in psycho-social functioning, between four subgroups of female adolescents who: (1) reported LOC eating with overvaluation; (2) reported LOC eating without overvaluation; (3) were obese individuals without LOC eating (obese controls); and (4) were normal weight without LOC eating (normal-weight controls). In view of the paucity of existing evidence, our only hypothesis was that adolescents with LOC eating who overvalued their weight or shape would have significantly higher levels of eating disorder psychopathology than obese controls and normal-weight controls.

## Method

### Study design and recruitment of participants

Participants were recruited as part of the *ACT Schools Mental Health Literacy Survey*, a cross-sectional study examining eating-disordered behaviour among secondary school students attending schools within the Australian Capital Territory (ACT) region of Australia (population of approx. 370,000 in 2012), which includes the city of Canberra [15]. The recruitment procedures have been detailed previously [16]. In brief, participants were recruited from 12 ACT schools, which varied in terms of type (Government, Independent, and Catholic), location and numbers of students at the school. The study was presented as an opportunity for schools to promote “mental health literacy” and no remuneration was provided. Consent to participate was required from both students and their parents. The study design and methods were approved by the Australian National University Human Research Ethics Committee (2011/573), the ACT Department of Education and Training (2011/00468-8) and the Catholic Education Office (R106903).

All students in classes selected for participation who attended class on the day(s) assigned for data collection were invited to complete a printed, self-report questionnaire, in their classrooms, under the supervision of a teacher and one or more members of the research team. The questionnaire included measures of eating disorder symptoms, general psychological distress, quality of life and basic demographic information. Body mass index (BMI: kg/m^2^) was calculated from self-reported height and weight [16]. Classification of weight status was based on the age-and-gender-specific BMI cut-points recommended by the Centers for Disease Control and Prevention (CDC) [17].

Completed questionnaires were received from 1749 students, a participation rate of 78.7% of all students invited to participate. Data for nine participants who were less than 12 years of age or greater than 18 years and a further 70 participants (4.0%) who were found to have unacceptably high levels of missing data or implausible data were excluded. The final sample therefore comprised of 1670 students aged 12–18 years. Of these, 1135 (68.0%) were female, 531 were male and 4 did not indicate their gender. The overrepresentation of female students, which reflected greater perceived relevance of the study material among the principals of all-girls schools and/or among the heads of faculties (e.g. psychology) in which female students were over-represented, was anticipated and was considered advantageous in addressing study aims that dictated relatively large numbers of participants with eating disorder symptoms [18]. The mean (SD) ages of male and female participants were, respectively, 14.85 (1.70) years and 15.51 (1.63) years. The sample comprised of 3.6% of all male secondary school students in the ACT in 2012 and 7.8% of all female secondary school students [19]. Reflecting the demographic profile of the ACT region, [20] the vast majority of participants were born in Australia (88.3%) and had English as a first language (90.4%).

Participants in the current study were the 1135 female adolescents. Male participants were excluded because the comparatively low prevalence of LOC eating among males precluded meaningful subgroups analysis. Although it would have been possible to combine male and female participants, this course was not taken because the goal of the current study was to extend prior research in women with BED and variants of BED to an adolescent population [7] and because, in the absence of any prior research on the status of overvaluation in males with BED or variants of BED, it was unclear how the inclusion of males might affect the findings.

### Study measures

#### Eating Disorder Examination Questionnaire (EDE-Q)

The EDE-Q is a 36-item self-report measure that assesses the occurrence and frequency of eating disorder features during the past 28 days [21]. A global score is derived from 22 items assessing core attitudinal features, namely, dietary restraint, concerns about eating, weight concern and shape concern. Scores on these items range from “0” to “6”, with higher scores indicating higher symptom levels [21]. Cronbach’s alpha for the global score in the current study sample was 0.97.

Remaining items of the EDE-Q assess the occurrence and frequency of specific eating disorder behaviours, namely, binge eating and the use of self-induced vomiting, laxative misuse and excessive exercise, as a means of controlling weight or shape. For the current study, an additional item assessing the occurrence of subjective binge eating episodes, namely, episodes of perceived overeating in which a loss of control is experienced but the amount of food consumed is not unusually large, was included. The inclusion of this item permitted the assessment of LOC eating, this being defined as the occurrence of objective and/or subjective binge eating episodes [16]. As in previous studies [22,23], minor changes to the wording of some EDE-Q items were made in order to ensure the suitability of the instrument for use in an adolescent population.

### Assessment of psychosocial functioning

Psychosocial functioning was assessed using measures of general psychological distress and quality of life as detailed below.

### Kessler (10-item) psychological distress scale (K-10)

The K-10 is a 10-item measure of general psychological distress developed for use as a screening instrument in epidemiological studies of mental disorders and widely used for this purpose [24,25]. In Australia, it is also used as an outcome measure for individuals treated in community mental health services and for routine population health monitoring [26]. Participants are required to indicate the frequency or occurrence of 10 symptoms of anxiety or depression during the past four weeks. Responses are scored on a five-point, Likert-type scale ranging from “none of the time” (“1”) to “all of the time” (“5”). Total scores therefore range from 10 to 50, with higher scores indicating higher levels of distress. The K-10 has very good psychometric properties and is suitable for use in both adult and adolescent populations [24,25,27,28]. Cronbach’s alpha for participants in the current study sample was 0.91.

### Pediatric quality of life inventory (15-item) (PedsQL™ 4.0 SF15)

The *PedsQL™4.0 SF15* is a 15-item, self-report, generic measure of quality of life developed specifically for use in paediatric populations [29,30]. Participants are asked how true each of a series of 15 statements were for them during the past four weeks, with items designed to address individuals’ perceived functioning in each of four domains: physical health (5 items); emotional well-being (4 items); social functioning (3 items); and academic (school) functioning (3 items). Responses are scored on a five-point, Likert-type scale ranging from “never” (“0”) to “always” (“4”), with higher scores indicating poorer perceived functioning in the domain concerned. In the current study, domain scores were calculated as mean scores of the specific items comprising each domain. The *PedsQL™4.0 SF15* has been found to be a reliable and valid measure of quality of life in young people, with psychometric properties comparable to those of the original (23-item) instrument [29,30]. Cronbach alphas in the current study sample ranged from 0.80 (physical health subscale) to 0.89 (total score).

### Creation of study subgroups

Consistent with the study aims, four subgroups of participants were created: (1) LOC eating with overvaluation; (2) LOC eating without overvaluation; (3) obese no LOC eating (obese controls); and (4) normal-weight no LOC eating (normal-weight controls).

Given that LOC eating, regardless of the amount of food consumed, indicates eating disorder and general psychopathology in youth [11,12], the current study, inline with prior research [14], focused on LOC eating. Consistent with DSM-5 criteria for binge frequency [31], “LOC eating” was defined as at least weekly LOC episodes during the past four weeks. Further, and also consistent with the DSM-5 criteria for BED, inclusion in the LOC subgroups required that regular LOC eating occurred in the absence of the regular use of extreme weight-control behaviours. In the absence of any agreed-upon operational definition of “regular extreme weight-control behaviours”, a conservative threshold, namely, twice per month, was employed in order to clearly distinguish participants in the LOC subgroups from individuals with a sub-threshold form of bulimia nervosa [32]. Thus, regular purging was defined as self-induced vomiting or misuse of laxatives or diuretics as a means of controlling weight or shape at least twice in the past 28 days. Regular extreme dietary restriction was defined, using an item of the EDE-Q, as going for long periods (8 or more waking hours) without eating anything at all as a means of controlling weight or shape more than 1–5 times in the past 28 days, and regular excessive exercise was defined as exercising in a “driven” or “compulsive” way as a means of controlling weight or shape at least twice in the past 28 days. These definitions have been used in previous, population-based studies of young women [7,33].

Participants with LOC eating were separated into those with and without overvaluation on the basis of responses to the two EDE-Q items that assess this construct, namely, “Over the past four weeks, how much has your *weight* influenced how you think about (judge) yourself as a person?” (“importance of weight”) and “Over the past four weeks, how much has your *shape* influenced how you think about (judge) yourself as a person?” (“importance of shape”). Consistent with previous research employing the EDE-Q within community samples [4,5], participants who scored 5 or 6 on either (or both) of these items were considered to have overvaluation. Scores of this magnitude indicate that self-evaluation was influenced by their weight or shape either “markedly” (score of “6”) or “more than moderately but less than markedly” (“5”). Items of the EDE-Q assessing overvaluation were omitted when comparing subgroups with respect to overall levels of eating disorder psychopathology as measured by the EDE-Q global score, thus creating a “revised” global score for the purpose of comparisons involving this outcome [7].

“Obese controls” were participants with a BMI above the 90th percentile for their age and sex [17] and who did not report any episodes of LOC eating during the past 28 days. “Normal-weight controls” were participants whose BMI was between the 5th and 85th percentile for their age and sex [17] and who reported no episodes of either LOC eating or extreme weight-control behaviours during the past 28 days. Participants who were underweight (BMI <5th percentile) were excluded from this subgroup. No attempt was made to exclude participants with overvaluation from the control groups since this would have detracted from the validity of comparisons between these groups and the LOC eating groups.

Of the 1,135 participants, 422 (37.2%) were classified as normal-weight controls, 36 (3.2%) were classified as obese controls, and 88 (7.8%) were classified as LOC eating. Among participants in the LOC eating subgroup, 30 (34.1%) overvalued their weight or shape while the remaining 58 participants (65.9%) did not have overvaluation.

### Statistical analysis

One-way analysis of variance (ANOVA) was used to compare scores on measures of eating disorder psychopathology (EDE-Q revised global score), general psychological distress (K-10), and quality of life (*PedsQL™4.0 SF15*) between groups. Post-hoc tests were used to identify the source of any statistically significant F values. In view of the small subgroup sizes and small number of outcome variables, no adjustment for multiple comparisons was employed. A significance level of 0.05 was used for all tests and all analysis was conducted using the IBM SPSS statistical software package (version 20.0).

## Results

As would be expected, differences between groups were observed with respect to BMI (*F*_(3,533)_ = 156.65, *p* < .05), whereas there were no differences between groups with respect to age, first language, country of birth, residential postcode or school type (all *p* > .05).

Results of the ANOVA appear in Table 1. As can be seen, significant differences between groups were observed for all outcome variables: EDE-Q (revised) global score (*F*_(3,542)_ = 70.16, *p* < .05), K-10 (*F*_(3,542)_ = 15.70, *p* < .05), and all subscales of the *PedsQL™4.0 SF15* (*F*_(3,541)_ = 20.71, *p* < .05). Post-hoc tests indicated that participants with LOC eating and overvaluation had higher EDE-Q global scores than women with LOC eating in the absence of overvaluation (*p* < .05), while scores on this measure did not differ between participants with LOC eating in the absence of overvaluation and obese controls (*p* > .05). Normal-weight participants had lower EDE-Q global scores than participants in all other groups (*p* < .05).

**Table 1:** **Mean (SD) scores on measures of eating disorder psychopathology (EDE-Q revised global score), general psychological distress (K-10), and quality of life (** ***PedsQL*** **™** ***4.0 SF15*** **) among study subgroups**

	**LOC eating with overvaluation of shape/weight (1)**	**LOC eating without overvaluation of shape/weight (2)**	**Obese controls (3)**	**Healthy controls (4)**			**Eta squared** ^**(i)**^	**Post hoc**
n	30	58	36	422				
	Mean (SD)	Mean (SD)	Mean (SD)	Mean (SD)	F	p		
Age	15.89 (1.37)	15.72 (1.72)	15.89 (1.56)	15.36 (1.70)	2.46	.062		
BMI	23.99 (4.89)	21.16 (3.46)	29.51 (3.83)	19.90 (2.21)	156.65	.000*		
	Mean (SE)	Mean (SE)	Mean (SE)	Mean (SE)	F	p		
EDE-Q revised global	3.31 (.20)	1.80 (.13)	1.80 (.20)	.94 (.05)	70.16	.000*	0.28	1 > 2,3 > 4
K10	27.23 (1.42)	24.40 (1.09)	20.64 (1.18)	19.73 (.34)	15.70	.000*	0.08	1, 2 > 4; 1 > 3
*PedsQL™* *4.0 SF15* subscales								
Emotional well-being	3.00 (.12)	2.63 (.10)	2.23 (.13)	2.20 (.04)	12.39	.000*	0.06	1, 2 > 4; 1 > 3
Social functioning	2.31 (.18)	2.13 (.13)	1.81 (.15)	1.66 (.03)	12.51	.000*	0.06	1, 2 > 4; 1 > 3
Academic functioning	3.31 (.19)	3.01 (.12)	2.60 (.18)	2.56 (.04)	9.54	.000*	0.05	1, 2 > 4; 1 > 3
Physical health	2.10 (.15)	2.10 (.12)	1.80 (.13)	1.60 (.03)	13.02	.000*	0.07	1, 2 > 4,
*PedsQL™* *4.0 SF15* Total	59.43 (2.51)	64.28 (2.09)	73.06 (2.69)	75.93 (.70)	20.71	.000*	0.10	1, 2 < 3, 4

*Significant *p* <0.05.

^i^eta squared effect size; 0.01 = small, 0.06 = medium, 0.14 = large [34].

There were no significant differences between LOC eating with overvaluation and LOC eating without overvaluation subgroups with respect to levels of general psychological distress or on any of the *PedsQL™4.0 SF15* subscales (all *p* > .05) and both of these groups had higher levels of distress, and higher scores on each of the *PedsQL™4.0 SF15* subscales, than normal-weight controls (all *p* < .05). Participants in the LOC eating with overvaluation subgroup had higher levels of distress, and significantly greater impairment in emotional, social and academic functioning, than obese controls (all *p* < .05), whereas scores on these measures did not differ between LOC eating without overvaluation and obese control subgroups (all *p* > .05). However both LOC eating subgroups with and without overvaluation reported significantly greater impairment on the *PedsQL™4.0 SF15* total than the obese control subgroup (*p* < .05), whilst there were no differences between participants in either of the LOC eating subgroups and obese control participants on the physical health subscale of the *PedsQL™4.0 SF15* (*p* > .05).

## Discussion

### Summary of main findings

The current study compared subgroups of female adolescents with LOC eating, with and without overvaluation of weight and/or shape, on measures of eating disorder psychopathology, general psychological distress, and quality of life. Subgroups of obese participants reporting no LOC eating (obese controls) and adolescents of normal weight not experiencing LOC eating (normal-weight controls) were included for comparison purposes. Participants with LOC eating and overvaluation had significantly higher levels of eating disorder psychopathology than all other subgroups, whereas participants with LOC eating in the absence of overvaluation did not differ from obese controls with respect to levels of eating disorder psychopathology. There were no significant differences between LOC eating with overvaluation and LOC eating without overvaluation subgroups on any of the other measures. Participants with LOC eating and overvaluation had significantly higher levels of distress and impairment in emotional, social and academic functioning than obese controls, whereas no such differences were observed between LOC eating without overvaluation and obese controls.

### Study implications

The finding that participants with LOC eating and overvaluation had significantly higher levels of eating disorder psychopathology than all other subgroups, when taken with the finding that participants in the LOC eating without overvaluation subgroup did not differ from obese controls in this regard, aligns with findings from previous research in adult [5-7] and adolescent populations [14]. Whilst overvaluation has been recognised as a distinct construct [6], high correlations between this construct and the broader construct of weight/shape concerns, as measured by the EDE-Q, exist [1,35]. The finding that the presence of overvaluation was associated with higher EDE-Q global scores, while notable, is not surprising, given significantly greater scores on the EDE-Q might reflect, in part, this association [35]. Nevertheless, the current findings in adolescents provides further evidence that overvaluation indicates disorder severity in terms of eating disorder psychopathology among individuals with BED and variants of BED [4,7]. Further, given that adolescents with LOC eating in the absence of overvaluation had comparable levels of eating disorder psychopathology to obese control participants, also in line with previous adult research [7], the current findings provide further evidence that LOC in the absence of overvaluation may not constitute a clinically significant pattern of behaviour.

Contrary to findings in both community and clinical samples of adult females with BED and variants of BED [4,7], but consistent with the findings of Goldschmidt and colleagues in adolescent females [14], no significant differences were observed between LOC eating subgroups on measures of psycho-social impairment, namely, general psychological distress and quality of life. However, there was evidence suggestive of such differences. Whereas participants with LOC eating and overvaluation had significantly higher levels of general psychological distress and impairment in social, psychological, and academic functioning than obese controls, there were no differences between participants with LOC eating in the absence of overvaluation and obese controls on any of these measures. These findings suggest that further investigation of the correlates of LOC eating with and without overvaluation in adolescents may be warranted.

At least two explanations for the lack of clear differences in levels of distress and disability between LOC eating subgroups among adolescents might be given, should this finding prove to be replicable. First, the concept of “overvaluation” may be more difficult for young people to grasp than for adults [10,14], making it more difficult to measure and differentiate between LOC eating with and without overvaluation. Second, evidence concerning the onset of overvaluation and its relationship to the onset of LOC eating and other eating disorder behaviours is lacking [36]. If overvaluation is associated with chronicity of overeating, then its influence on young people with LOC eating might only become apparent later in life. This interpretation would be consistent with the observation, in the current study and in Goldschmidt et al.’s study [14], that the presence of overvaluation among young people with LOC eating indicates greater severity in terms of eating disorder psychopathology but not in terms of distress and disability. Further population-based research, employing a prospective study design and, if possible, interview assessment of key constructs will be needed to elucidate these different possible interpretations.

### Study limitations

At least four limitations of the current study methods should be noted. First, all variables were assessed using self-report measures. While self-report assessment of psycho-social functioning is unlikely to be problematic, limitations inherent in the self-report assessment of eating-disordered behaviour are well known [21,37,38]. In particular, the occurrence and/or frequency of binge eating may be overestimated when using self-report assessment and self-report assessment of loss of control over eating may be unreliable when the amount of food consumed is not unusually large [21,37,38]. Further, whereas high correlations between self-report and interview assessment of weight/shape concerns have been observed in several studies, little is known about the validity of self-report assessment of the more specific construct of overvaluation [21,37,38]. A study of young adult women supported the validity of self-report assessment of overvaluation, when compared with interview assessment [37], but evidence in adolescents is lacking [38]. It should also be noted that the EDE-Q assesses LOC eating and other behaviours over the past 28 days, as opposed to the 3-month duration specified in DSM-5 criteria for BED [31]. It is possible that different findings would have been observed had interview assessment and/or a 3-month time frame been employed.

Second, sample size for the LOC eating and obese control subgroups (n = 30, n = 58, n = 36) was relatively small. Hence, the failure to detect significant differences between these groups may reflect, in part, a lack of statistical power. The finding that there were significant differences between LOC eating with overvaluation and obese control participants on measures of distress and quality of life, but no such differences between LOC eating without overvaluation and obese control participants, is consistent with this interpretation.

Third, the cross-sectional design of the current study precludes any inferences as to the correlates of LOC eating with and without overvaluation over time. Research employing a prospective study design is needed to examine issues such as the utility of overvaluation in predicting outcomes among adolescents with LOC eating, and its temporal stability.

Finally, as in previous community-based studies, only females were included in the current study. Although very large sample sizes would be needed to examine the status of overvaluation among adolescent males with LOC eating in a community sample, research of this kind would be of interest given that LOC eating may be relatively common in boys [16].

Strengths of the current study include the recruitment of participants from a large, population-based sample, high participation rates, the inclusion of obese control and normal-weight control groups, and the inclusion of established measures of eating disorder psychopathology, general psychological distress and quality of life.

## Conclusion

The results provide further evidence that the presence of overvaluation among adolescents with LOC eating indicates a more severe disorder in terms of eating disorder psychopathology, whereas overvaluation may not indicate distress and disability as clearly as it does among adults with BED and variants of BED.

## Abbreviations

BED: Binge eating disorder
LOC eating: Loss of control eating
ACT: The Australian Capital Territory
